# Application and Evaluation of SPD Based Logistics Management Model for Medical Consumables in Clinical Nursing Departments

**Published:** 2019-08

**Authors:** Chai YANG, Wei GU, Tongzhu LIU

**Affiliations:** 1. School of Management, Hefei University of Technology, Hefei, 230009, PR China; 2. The First Affiliated Hospital of USTC, Division of Life Sciences and Medicine, University of Science and Technology of China, Hefei, 230031, China

**Keywords:** Medical consumables, Hospitals, SPD, Clinical care, Clinical nursing department

## Abstract

**Background:**

Supply, processing, and distribution (SPD) model is sparingly used in hospitals in China. We evaluated its effects on the management efficiency, quality control, and operating costs of medical consumables (MCs) in the clinical nursing surroundings in a single Chinese hospital-Anhui Provincial Hospital from 2014 to 2015.

**Methods:**

Amount-based packages (ABP) and procedure-based packages (PBP) models were created. They were introduced the use of quick response (QR) code scanning for using in clinical nursing departments (CNDs). Questionnaires were prepared by referring to previous literature and using Delphi method repeatedly, further discussed and formalized. Partial results of the formal questionnaire were analyzed using SPSS.

**Results:**

Frequency of MCs claims reduced without any requirements of MCs in 70% of CNDs. Average time spent on the inventory per week decreased and the time required to procure MCs reduced. Moreover, the average satisfaction score with MCs management increased, reaching 100%. Average space occupied by MCs decreased significantly, reducing by 1.2444m3. Overall, 100% of the respondents concluded that the management of MCs improved effectively and the inventory turnover rate had accelerated. The cost of MCs decreased by 15% with more than 10% increase in in-hospital amount, and the average daily cost of MCs also showed decrease.

**Conclusion:**

SPD can improve the efficiency of MCs management in CNDs, reducing medical risks and disputes, saving hospital operating costs, and decreasing capital occupation.

## Introduction

Implementation of advanced material management concepts in a few hospitals has been beneficial (1,2). For instance, just-in-time (JIT) procurement and zero inventory approach for medical consumables (MCs) management (3); alterations to supply chain management for materials management (4); hospital logistics management information systems for information sharing and integration (5). Supply, processing and distribution (SPD) model is primarily concerned with the medical materials in a hospital. This model employs logistics information technology as a tool, subcontracts or centralizes the management of MCs logistics, hence, ensuring an increased efficiency of the hospital (6). In the 1990s, Japan introduced the medical industry to JIT management technology, and gradually developed SPD management model (7,8). As the SPD model is suited to the current management needs of Chinese hospitals, its increased usage has great potential to achieve higher efficiency in hospital management. While overall in China mainland, only six hospitals to date have integrated the SPD model into their hospital management system, just focusing on drug management (9, 10).

Traditional MCs management is now facing many problems including the models cause wastage of resources in the MCs logistics chain, seriously influencing increase in medical expenses and the risk of medical accidents in hospitals (11–14).

The purpose of this study was to evaluate the effects of SPD model on management efficiency, quality control, and operating costs of MCs in the clinical nursing departments (CNDs) in a single Chinese hospital to improve product management issues faced in hospitals.

## Materials and Methods

### Study object

This study was performed in Anhui Provincial Hospital, Hefei, PR China, a large provincial general hospital with 4321 open beds and 61 CNDs. In 2016, total number of emergency and outpatients were 3251,000, total number of in-patients were 175,000, total number of surgeries performed were 63,000, and total amount of annual MCs were over $9 million. We selected 30 CNDs in our hospital to implement SPD model.

The study was approved by the hospital’s Ethics Committee and all participants provided written informed consent after completing the study.

This large hospital was selected based on the following key research criteria:

- It is necessary to select a hospital where the management of MCs for the implementation of SPD model plays a core role in hospital management, such that research activities would be ongoing continuously without any suspension. This hospital is the first institution in China to apply the SPD management model to MCs (15) also regarded as a benchmarking enterprise in the supply chain management of Chinese hospitals;
- Application of SPD model requires a large persuasive sample database. According to our hospital’s total number of emergency procedures and the statistics of admissions, this hospital has the ability to provide sufficient amount of detailed data for this research. The extracted information from this massive data would be highly objective and widely applicable, hence having the potential to provide guidance to other hospitals. In addition, we selected CNDs for evaluating this model because the most important aspect of hospital supply chain performance is treatment efficacy without any delays in treatment and clinical care processing time (16).

### Management methods

#### Operation mode

Suppliers to all of the clinical care departments were required to comply with certain criteria, and to follow a “fixed” delivery mode for commonly used medical supplies, including amount-based packages (ABP) in normal clinical departments and procedure-based packages (PBP) in operating rooms.

*ABP*: Based on previous literature and practices of doctors and nurses, the CNDs quantitatively estimated and defined the amount of MCs required for one week as a “fixed” number.
*PBP*: based on specific diseases and different clinical departments.

**Fig. 1:**
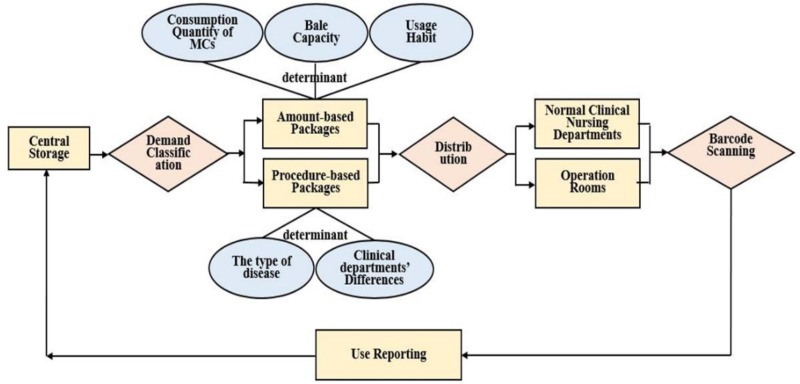
“Fixed” delivery mode for MCs

Some “fixed” numbers were lower than the quantities supplied in whole-box packaging, therefore, the suppliers were required to split and re-package MCs. (“Fixed” delivery mode for MCs shown in Fig. 1).

##### Implementation of quick response (QR) code management for MCs:

QR code is a type of advanced logistics identification technology (17). The information of this code can contain is flexible depending on the specific departmental requirements. In this hospital, a dedicated QR code label (including the item name, specification, manufacturer, date of manufacture, expiry date, and quantity) was affixed to all MCs and split items were delivered by the suppliers. These suppliers were requested to dispatch supplies to the clinical care department’s secondary warehouse only after this code was scanned, followed by acknowledgement of receipt by the hospital. Healthcare workers teared off the QR code and pasted it onto a unified collection form when they used the MCs. Each week, the supplier replenished the MCs, recycled the collection form, and re-scanned the QR code. Statistics and analysis of existing weekly consumption of MCs by the clinical care department were generated, and these data provided the basis for replenishment in the next cycle. To aid the management of the clinical care department, the supplier copied the form and handed it to them for their records.

### Methods for data collection and analysis

Data is derived from system extraction and questionnaire survey.

- A descriptive statistical method was used to derive data from the SPD model system for comparison with data from the hospital business operating system (HBOS) before the model was introduced. Data were extracted on the frequency of materials management input by the CNDs. comparisons were made for the periods July to Oct 2014 (before the introduction of the new model), and Jul to Oct 2015 (after the model’s introduction).
- Since some data was not recorded by the system, it was necessary to conduct a questionnaire survey of the personnel using the system.

Here are the steps to create a questionnaire.

**Fig. 2:**
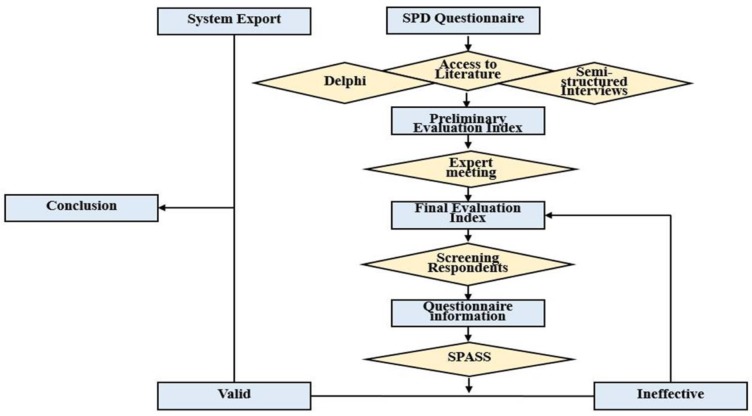
Data extraction and analysis model

Step 1: *Devise a questionnaire entry pool*. By reviewing existing literature (16, 18–20) and interviewing experts, 14 questions with three subparts was developed.
Step 2: *Form the original questionnaire*. First, choose consultant. Second, prepare a questionnaire. The questionnaire included preamble, subject, and expert information questionnaire. Third, consultation method. A judgment sampling method was adopted to invite 20 experts internal and external of the hospital on the initial questionnaire for two rounds of Delphi consulting and the two rounds of consultation were for the same object library members. The results of the second round completed the investigation. Fourth, statistical analysis of data was performed.
Step 3: *Confirmation of Questionnaire Survey*. The following indicators were selected by the experts in the hospital (Hospital Director, directors of the CNDs, head nurses and senior researcher of medical equipment) and were confirmed for inclusion in the questionnaire: (i) time taken for clinical care department inventory and weekly sorting; (ii) time is taken to obtain consumables through the electronic information system; (iii) satisfaction with consumables management work; (iv) space occupied by consumables; (v) range of consumables used.
Step 4: Data analysis. SPSS 17.0 (Chicago, IL, USA) software was used to perform statistical analysis of other indicators. Due to the small number of clinical departments involved in the investigation, the measurement data was used to assess whether differences were significant (21), and statistical significance was considered at *P*<0.05. Data extraction and analysis has been illustrated as a model in Fig. 2.

## Results

We collected and analyzed the relevant data. The data analysis results are shown in details in Table 1 and each figure. All the analyzed data were significantly different between the two periods (*P*<0.05). Space occupied by supplies reduced after the introduction of the new model. The mean occupancy by consumables in the former system was 4.9447 m^3^ (SEM= ±0.01966), and when we used the SPD model, it reduced to 3.7003 m^3^ (SEM= ±0.01334) (Table 1). The secondary warehouses used goods frames and boxes to place the consumables, allowing unified management and collation of the goods. Consumables were labeled and arranged that greatly enhance the cleanliness and appearance of the warehouses. Under the new model, materials were removed from their packaging, significantly reducing the average space occupied by 1.2444m^3^, which released about 25.92% of the space.

**Table 1:** Comparison of the relevant indexes of supplies management before and after SPD application (X ± S)

***Project***	***Before (Mean±SEM)***	***After (Mean±SEM)***	***t***	***P***
Collection frequency (time/month)	10.40 ± 0.59	0.20 ± 0.15	19.308	<0.001
Weekly inventory and sorting time (minutes)	51.00 ± 5.62	2.83 ± 0.59	9.319	<0.001
Application time (minutes)	12.00 ± 0.86	3.97 ± 0.58	10.650	<0.001
Consumables management satisfaction (points)	2.83 ± 0.15	4.87 ±0.06	−14.56	<0.001
Consumables occupancy space (m^3^)	4.94 ± 0.02	3.70 ± 0.01	70.15	<0.001

**Fig. 3:**
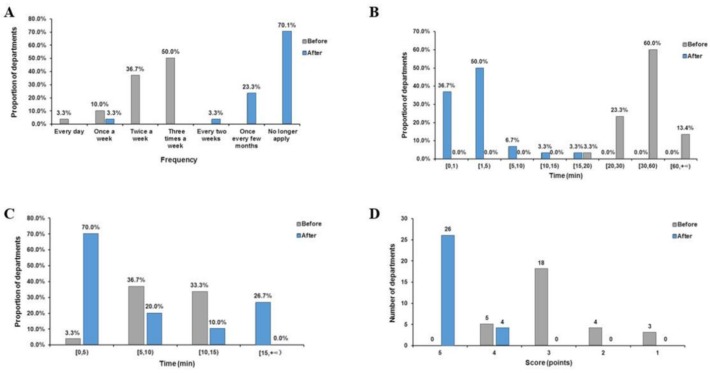
(A) Frequency of ordering consumables in the clinical nursing departments before and after introduction of the SPD model, (B) Weekly clinical care department inventory and goods sorting time before and after introduction, (C) Application time before and after introduction, (D) Consumables management satisfaction before and after introduction

The frequency with which staff ordered medical consumables decreased significantly after the introduction of the new model (Fig. 3A). Before introducing the model, the mean frequency value was 10.4 time/month (SEM= ±0.594), which reduced to 0.2 time/month (SEM= ±0.147) after the implementation of this model (Table 1). Moreover, the orders made by staff significantly reduced in frequency, with 70% of the clinical care departments making no new orders for MCs, and the remaining departments reporting very low ordering frequency through the electronic application system. The departments also reported borrowing from other clinical care departments when necessary.

The time devoted to item storage was greatly reduced after the introduction of the new model (Fig. 3B). Before the model was introduced, the mean time taken to inventory and sort materials was 51 min/week (SEM= ±5.619). After the model was introduced, it reduced to 2.83 min/week (SEM= ±0.591) (Table 1). Nearly 96.7% of clinical care departments needed more than 20 min to organize supplies under the old system, but after the introduction of the new model, nearly 86.7% reported time that was less than 5 min. These results reflect the orderly placement of supplies and the utility of the goods storage records. These changes have made the storage of goods a simple and coherent process. Clinical nurses now did not need to spend a lot of time accounting for and organizing goods and have more time to devote to their patients.

The time required to obtain consumables through the electronic information system was greatly reduced after the new model was introduced (Fig. 3C). The mean time spent before the introduction of the model was 12 min/day (SEM= ±0.855), but it reduced to 3.97min/day (SEM= ±0.580) after the introduction of the model (Table 1). The traditional way of claiming consumables was to use the office automation (OA) system to fill in a requisition form. However, because the OA system was not a purpose-designed medical consumables management software, it was often necessary to insert artificial information when filling in the application details, for example, the batch number, name of the manufacturer, or quantity, which was all very time-consuming. Since the introduction of the new SPD application system, this accessory information about consumables could be conveniently accessed, allowing faster completion of applications. According to our investigation, the time taken by CNDs to complete the traditional application was more than 10 min, while now 70% of them can complete the electronic SPD application in 5 min.

The application of SPD improved the satisfaction of nurses (Fig. 3D). The average MCs management satisfaction score was 2.83 points (SEM= ±0.152) under the old system, but it increased to 4.87 points (SEM= ±0.063) with the new model (The perfect score is 5 points) (Table 1). After applying the SPD system, the satisfaction rate increased significantly, from 76.7% to 100%. In order to facilitate comparison, we also used 10-point Likert scale (ranging from “completely disagrees” to “completely agrees”). According to the survey results, the satisfaction assessment was an average of 9.683 points.

In the open question survey, we also received other descriptive feedback and data feedback, these were: (i) the inventory turnover rate was reduced from approximately 15 d/time to approximately 3 d/time; (ii) the average cost of MCs used daily decreased from $30.19 to $ 23.96 per bed; (iii) all CNDs used the new model can effectively carry out the validity of MCs management; (iv) on the basis of more than 10% increase in discharge from the hospital, the cost of MCs decreased by 15%, effectively reducing the CNDs MCs costs; (v) the management of the QR code attached to the disease type and charging project effectively curbed the waste of MCs from the transfer to the department; (vi) all CNDs agree that the introduction of new model is conducive to the extraction and statistical analysis of consumables information.

## Discussion

We have conducted a series of analyses on the research and development of SPD in Anhui Provincial Hospital (15, 22) including the current work. Through the method of data export and questionnaire, we determined that in comparison with other supply chain models, SPD model can: (i) effectively improve the management efficiency of MCs in CNDs by effectively controlling the cost, reducing capital occupation, space and improving quality control. The storage environment was improved, and consumables were registered and easy to find. Compared with 25.92% space reduction, they were able to release 8% of the capacity of their sterile supply room (23), implying that the consumption space under SPD management was obviously smaller than the same conditions under the implementation of Kanban management.

(ii) In particular, SPD system uses the appropriate logistics equipment, combined with ABP and PBP to provide active, timely and professional logistics and distribution. It not only reduced the nurse’s regular inventory and regular application of the consumption of materials (The similar study was also conducted at the Shanghai Oriental Hospital - a big size hospital with 3280 beds (24), the application time there was reduced by about 50 h per month after the model was introduced.) but also reduced the handling of high-volume supplies and work put in for inspection. In a similar work, a sustainable web-based PCIP was created for improving supply chain management of medications (25). Annual average drug application frequency was about 344.92 times, while in the SPD mode, the annual application frequency was only 2.4 times. This completely reflects the superiority of the integrated supply and distribution features of the SPD model, but also the timeliness of suppliers in supplementing supplies, and the benefits of consulting with these suppliers to develop rational rules. This saved the time put in by doctors and nurses in physically managing supplies, thereby increasing their supplies management work satisfaction, since they can now use this time for clinical work, which ultimately improved patient quality of service, the satisfaction (9.683 points) was much higher than the 6.94 points of similar medical evaluation results obtained by Kanban’s management in Hospital Universitario Virgen Macarena y Área (AHVM), located in Seville (26). (iii) Before introducing the SPD mode, only high value of the MCs was sold on credit. After the model was introduced, all the MCs were sold on credit. The average monthly occupying funds of normal MCs decreased by $30,000. (iv) The management of the QR code attached to the disease type and charging project effectively curbed the waste of MCs from the transfer to the department. (v) Convenience for data statistic. The new model made the warehouse record of the MCs information electronically played an important role in MCs consumption and tracking. These basic data provided sufficient information for various management and research to be done in the future. (vi) QR code technology played an important role in the traceability of consumables. Consumables could be tracked from purchase to use, which could help improve clinical safety, reduce medical risks and disputes.

Although scholars have comprehensive theoretical knowledge of similar approach, there has been very limited discussion and research on the specific effects of its implementation at the clinical level (27, 28). For instance, we have previously shown the use of Business intelligence (BI) system in supply chain data that can provide corresponding intelligent services (22), however, for user feedback, system data from outside cannot be obtained through this system. This is not only true for the SPD model, but for many supply chain models in the medical field. In other aspects, for instance, Kanban management can complete the automatic replenishment (26), but does not mention ABP and PBP customization issues, and is still not conducive to control cost. The lack of a precise and holistic approach is observed in the composite model of Six Sigma, a lean methodology for the entire hospital operations (29). It displays a lack of feedback to decision makers and potential users that is not conducive for the improvement of hospital standards. Therefore, our research can prove that the SPD model has stronger advantages, which can help the hospital to improve management level effectively.

While our analyses show promising results, it still faces some challenges. At present, only a handful of hospitals have introduced the SPD model for their logistics supply chain management, but the scientific credibility and universality of this model has the potential for its expansion in the future. Next, we will promote the model in other hospitals in China, in other developing countries and even some developed countries so that they could improve the management of MCs in hospitals and this will take a long time.

However, our study may have some limitations including sample selection only in China’s large-scale comprehensive hospital and uncertainty of applicability of these results in other departments of the hospital. Data extraction based on the SPD is needed from various information systems in hospitals. The limited collaboration between these systems can also limit the capability of testing this model and hence needs further improvement.

## Conclusion

Here, we set out to evaluate the effects of SPD model for MCs in CNDs. The SPD model can effectively help CNDs to manage MCs. The time to organize the collection is reduced, inventory decreased significantly, the warehouse became clean and orderly; the frequency and time spent on the claim fell significantly. Altogether, this economized on manpower; provided database for all management and MCs traceability; and liberated medical staff from the cumbersome business of material management.

## Ethical considerations

Ethical issues (including plagiarism, informed consent, misconduct, data fabrication and/or falsification, double publication and/or submission, redundancy, etc.) have been completely considered by the authors.
